# Performance of Host-Races of the Fruit Fly, *Tephritis conura* on a Derived Host Plant, the Cabbage Thistle *Cirsium oleraceum*: Implications for the Original Host Shift

**DOI:** 10.1673/031.008.6601

**Published:** 2008-10-29

**Authors:** Thorsten Diegisser, Jes Johannesen, Alfred Seitz

**Affiliations:** Institut für Zoologie, Abteilung für Ökologie, Universität Mainz, Saarstrasse 21, D-55099 Mainz, Germany

**Keywords:** alternative host, *Cirsium heterophyllum*, fitness, oviposition, performance, speciation

## Abstract

The thistle-infesting fruit fly *Tephritis conura* Loew (Diptera: Tephritidae) forms host races on the melancholy thistle, *Cirsium hetewphyllum* (L.) Hill (Asterales: Asteraceae) and the cabbage thistle, *Cirsium olemceum* (L.). Scop. Previous research indicates that the host shift occurred from *C. hetewphyllum* to *C. oleraceum.* In this paper we address whether the host shift involved physiological adaptations by studying oviposition acceptance and survival of the two host races on the derived host *C. oleraceum.* Performance differed significantly between host races. *T. conura* originating from *C. oleraceum* produced adults in 75% of all egg-laying trials in contrast to only 6.6% in *T. conura* originating from *C. hetewphyllum.* Population fitness components measured as a function of life-stage was linear decreasing for *T. conura* on *C. oleraceum* but stepwise for *T. conura on C. heterophyllum*. Low performance of *T. conura* on *C. hetewphyllum* was determined by low plant acceptance and high mortality during the larval stage, whereas hatching (at least one larva per batch) and pupae survival were not affected.

## Introduction

Host-specific insects are estimated to represent 25–40% of all animal species (Zwölfer 1975; Bush and Butlin 2004). It has been proposed that many of these phytophagous insects have arisen via host shifts to a new plant with subsequent specialization (Bush 1975). The colonization of a new host theoretically requires only a genetic change in host preference (Dres and Mallet 2002). However, adaptations to different host-plant phenologies (e.g. Craig et al. 1993; Feder et al. 1997; Thomas et al. 2003) and host plant physiology may be required. Plants that differ in the amount of secondary metabolites (Roitberg and Isman 1992) and nutritional value (Häggström and Larsson 1995) can result in reduced growth and survival of the feeding larvae (Sharma and Norris 1991). Host-dependent larval performance has been demonstrated in numerous studies (e.g. Via 1991; Craig et al. 1993, 1997; Katagura and Hosogai 1994), indicating that physiological adaptations play an important role in the course of a host shift. Distinguishing the causes responsible for divergence is important for reconstructing how host races evolve. If larvae develop on different plants equally well, only a change in host preference might have been the critical requirement to initiate the host shift. However, if insects from the ancestral host population were not physiologically adapted to the novel host, the initial shift would have been maladaptive but after adaptation perhaps physiological adaptations became a prerequisite for diversification.

A promising candidate for host-mediated speciation is the thistle-infesting fruit fly *Tephritis conura* Loew (Diptera: Tephritidae), which infests flower heads of several *Cirsium* (Asterales: Asteraceae: Cardueae) species (Zwölfer 1988) including *Cirsium heterophyllum* (L.) Hill and *Cirsium oleraceum* (L.) Scop. Genetic analyses have shown that gene flow among *T. conura* infesting *C. heterophyllum* or *C. oleraceum* is severely restricted (Seitz and Komma 1984; Diegisser et al. 2006a,b) and that host-specific morphological adaptations exist (Diegisser et al. 2007). In hostchoice experiments, *T. conura* significantly preferred to fly to and to stay on the host-plant species from which they had emerged (Komma 1990; Diegisser 2005). As *T. conura* mate exclusively on their host, host preferences cause assortative mating, which is a prerequisite for further differentiation. Preliminary experiments could show that *T. conura* have adapted behaviourally to the phenology of host plants; in the lab, *T. conura* on *C. heterophyllum* become significantly earlier sexually active than *T. conura* on *C. oleraceum* after hibernation, which coincides with plant phenology in the field (Diegisser 2005).

These findings provide evidence for two host races, i.e. partially reproductively isolated populations due to differential host specialization. Phylogeographic mtDNA data strongly suggest that *C. heterophyllum* is the ancestral host, from which flies shifted to *C. oleraceum* (Diegisser et al. 2006a). No study has yet examined whether *T. conura*'*s* host shift from *C. heterophyllum* to *C. oleraceum* has involved physiological adaptations. In this paper, the results of an oviposition experiment are presented which estimates acceptance and performance of both *T. conura* host races on the derived host, *C. oleraceum.* By doing so, it is concluded that *T. conura* from the ancestral C. *heterophyllum* population may have adapted physiologically to *C. oleraceum* in the course of the host shift.

## Materials and Methods

### Biology of T. conura

The univoltine *T. conura* completes larval development in flower heads of several *Cirsium* species. As soon as the host plants start developing flower buds in late spring, *T. conura* imagoes reappear from unknown hibernation sites. After mating, which occurs exclusively on the host plants, females lay egg batches in young buds. Depending on the host plant, eggs are laid from the middle of May (*C. hetewphyllum*) to the end of June (*C. oleraceum*)*.* Within the developing flower heads, larvae feed on the florets (first instar) and the receptacle area (second and third instar). Larvae pupate about three weeks after oviposition; imagoes emerge three weeks after pupation.

### Oviposition experiment

Rosettes were collected for experiments from *C. oleraceum* in the Bavarian Fichtelgebirge (Eimersmühle, N49°53.092/E011°36.975, 396m asl) at the beginning of May 2003. All plants (N = 100) were potted in 5L flowerpots and kept in a greenhouse. In the beginning of June, the rosettes started developing buds that could be used for oviposition experiments. Buds were formed on new stem growth in the rosette. In general, 5–10 apical buds developed together in a tight cluster. *C. oleraceum* produces buds continuously throughout the flowering season. Continuous bud development ensured that all flies were offered buds in the same phenological stage and of the same size.

*T. conura* were collected in the field as soon as they re-colonized the host populations after hibernation. Female *T. conura* were collected from *C. hetewphyllum* at Bischofsgrün (N50°03.479/E011°46.588, 630m asl) at the end of May 2003. Female *T. conura* were collected from *C. oleracum* at Eimersmühle (see above) in the middle of June 2003. Preliminary experiments in 2002 showed that *T. conura* have already mated when first found in the field and that females kept in a cage would oviposit into the cage's gauze if no host plant was offered. These flies survived for +30 days after being caught.

*T. conura* infesting *C. heterophyllum* were collected about 10 days before the first *C. oleraceum* in the lab were ready for experimental trials while *T. conura* infesting *C. oleraceum* were caught two weeks later when plants in the lab were ready for experiments (see above). To standardize the experiments between the host races, all flies were kept in the lab for an average of 12 days before starting the experiments.

Experiments were performed by offering single females apical buds of a *C. oleraceum* plant. Flies and buds were enclosed in a gauze bag. Buds had a diameter between 6–11 mm diameter, which is the size of buds accepted by both host races in the field. Previous lab experiments showed that *T. conura* adapted to *C. heterophyllum* or *C. oleraceum* accept buds between 5.8–13.7mm and 3.4–11mm diameter, respectively (Eschenbacher 1982; Romstöck 1982). Buds are enveloped by small leaflets, which have to be pierced by the fly's ovipositor in order to deposit the eggs into the flower head. As a consequence, oviposition attempts can be inferred from tiny dark holes in the enveloping leaflets. Each plant was checked daily for such holes. If punctures were detected, females were removed two days later. If no holes had been found after a week, the trial was terminated.

Four weeks after removal of flies, flower heads were cut and placed separately in small jars covered with gauze. As *T. conura* pupates within three weeks after oviposition (see above) this transfer most likely did not affect the development of flies within a flower head. The jars were checked daily for emerging flies. After four weeks in ajar, each flower head was dissected to check for non-emergence of larvae and pupae. The total time involved from oviposition to flower-head dissection was eight weeks. This time period is sufficient to ensure quantitative emergence because *T. conura* completes larval development within six weeks.

The experiment estimated population fitness components at four life-stages. (1) Oviposition attempts, defined as the number of flies accepting buds. (2) The number of flies successfully producing larvae. The presence of larvae can easily be inferred from the damaged brownish/dark-brown colour of the receptacle area. Damage is not seen in non-viable oviposition attempts. (3) Survival from larvae to pupae was quantified as the number of flies producing pupae, and the number of pupae. (4) Survival from pupa to adult was estimated as the number of females producing adults and as the difference between emerging adults and the number of pupae per flower head. Individual mortality in the egg and larval stages were not estimated because the number of eggs and larvae could not be determined reliably without destructive or subsampling of the host materials. (2) and (3) are therefore qualitative estimates of a fly's ability to produce at least one surviving offspring per egg batch. Between-species plant acceptance and survival were tested with independent 2 × 2 contingency table χ″^2^ tests.

## Results

**Figure 1.**
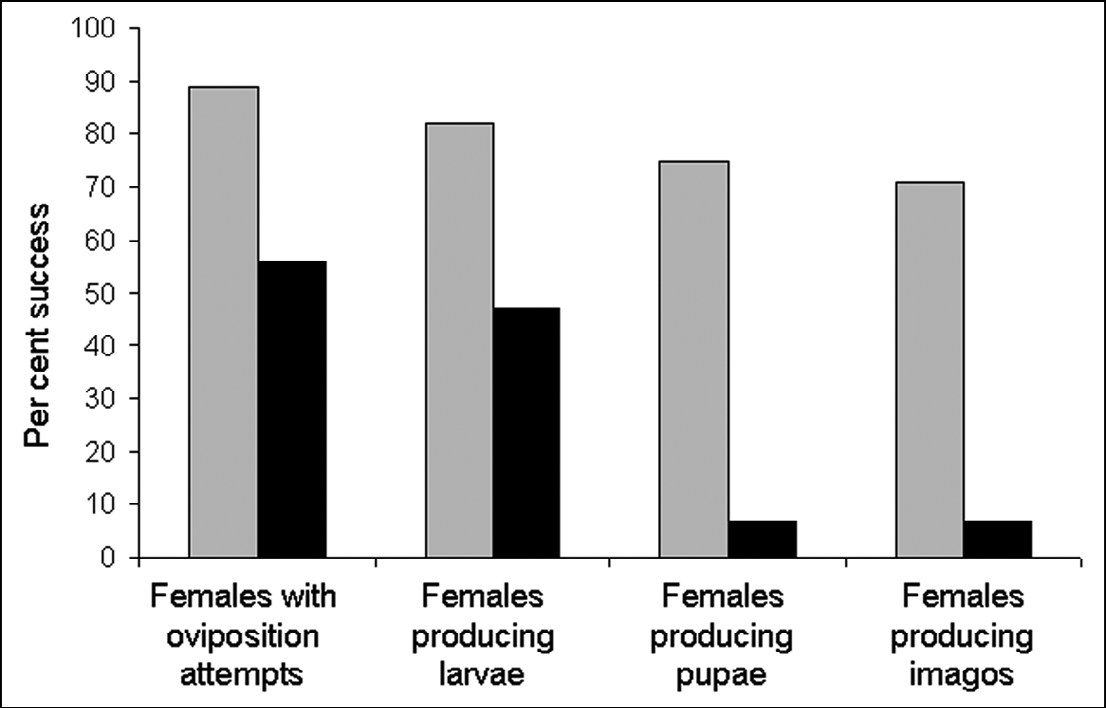
The percentage of *Tephritis* conura adapted to *Cirsium oleraceum* (N = 28, grey bars) and *T. conura* adapted to *C. heterophyllum* flies (N = 45, black bars) accepting *C. oleraceum* inflorescences for oviposition and producing offspring depicted as a function of proceeding life-stage. Note that survival decreases linearly for *T. conura* adapted to *C. oleraceum* and decreases step-wise for *T. conura* adapted to *C. heterophyllum*.

Acceptance of *C. oleraceum* flower buds differed between host races. Population fitness components measured as a function of developmental stage decreased linearly for *T. conura* adapted to *C. oleraceum* but decreased stepwise for *T. conura* adapted to *C. heterophyllum* (Figure 1). 89.3% of *T. conura* adapted to *C. oleraceum* (25 out of 28) and only 55.6% *T. conura* adapted to *C. heterophyllum* (25 out of 45) attempted to oviposit within a week, but the difference was not significant (χ″^2^ = 1.20, d.f. = 1, P = 0.27) (Figure 1). For those flies that attempted to oviposit, signs of larval parasitation were very similar: *T. conura* adapted to *C. oleraceum*, 92% (N = 23); *T. conura* adapted to *C. heterophyllum* flies, 84% (N = 21) (χ″^2^ = 0.002, d.f. = 1, P = 0.99). Mortality in the larval stage was significantly higher for *T. conura* adapted to *C. heterophyllum*, where larvae from only three out of 21 flowers (14%) reached the pupal stage compared to 21 out of 23 (91%) parasitized by *T. conura* adapted to *C. oleraceum* (χ″^2^ = 6.97, d.f. = 1, P < 0.01) (Figure 1). The mean number of pupae per flower head for *T. conura* adapted to *C. oleraceum* was 9.05 (± 6.78) but only 4.33 (± 4.04) for *T. conura* adapted to *C. heterophyllum.* The mean number of emerging imagoes per flower head for *T. conura* adapted to *C. oleraceum* was 8.05 (± 6.67), while the three flowers containing pupae of *T. conura* adapted to *C. heterophyllum* produced an average of 3.67 imagoes (one fly produced 9 imagoes, the two other produced only one imago each, see Table 1). However, if larvae of either host-race reached the pupae stage, pupae developed into imagoes with equal probability (for the *C. oleraceum* host race, per flower 95.2%, per individual: 85.2%; for the *C. heterophyllum* host race, per flower 100%, per individual: 84.6%). In total, 75.0% of *T. conura* adapted to *C. oleraceum* produced at least one imago compared to only 6.6% of *T. conura* adapted to *C. heterophyllum* flies (χ″^2^ = 15.54, d.f. = 1, P < 0.0001).

## Discussion

The oviposition experiment revealed that *T. conura* adapted to *C. heterophyllum* exploited the derived host *C. oleraceum* significantly less well than *T. conura* adapted to *C. oleraceum.* This was determined by unwillingness to accept *C. oleraceum* buds and by high mortality during the larval stage, whereas survival of at least one egg to hatching after oviposition attempts and pupae to imago were not affected. This shows that *T. conura* adapted to *C. oleraceum* were significantly better adapted than *T. conura* adapted to *C. heterophyllum* flies to the larval environment provided by *C. oleraceum* flower heads. The two stages not affected by mortality can be viewed as stages that are not directly involved in interactions with the host plant. Different performance may be biased if the host races responded differently to the induced oviposition delay or if the developmental buffer time was not long enough to ensure survival of *T. conura* adapted to *C. heterophyllum* on the novel host. If these factors did influence the results, they also imply that the host races will respond differently to conditions experienced in the field; conditions that will be of advantageous to the *T. conura* adapted to *C. oleraceum* host race.

The inclusion of a novel host may involve “host-range expansion” where an insect population retains the ability to utilise old hosts (Hare 1990). Alternatively, the insect may be unable to adapt simultaneously to both, resulting in a “host shift” with host plant-associated fitness tradeoffs (Via, 1990). To evaluate which of these performance scenarios influence the evolution of *T. conura* host-races, reciprocal experiments are needed. Regrettably, despite great efforts, we have not been able to bring *C. heterophyllum* rosettes to develop buds in the greenhouse to perform the reciprocal experiments (for details see Diegisser 2005). Attraction tests (Diegisser 2005) showed that host races are attracted significantly more to their own host plants, implying that acceptance of *C. heterophyllum* flower heads will be significantly lower for *T. conura* adapted to *C. oleraceum.* Data for naïve flies showed that female *T. conura* adapted to *C. oleraceum* preferred their own plants, which required *C. oleraceum* to become sexually active (Diegisser 2005). This suggests that female *T. conura* adapted to *C. oleraceum* will not accept *C. heterophyllum* in the field.

For the life-stage with performance differences within the flower head, larvae to pupae, the field evidence shows that heterophyllum flies are most fit on *C. heterophyllum*, but, unfortunately, no field data is available for the reciprocal mortality of *T. conura* larvae adapted to *C. oleraceum* on *C. heterophyllum.* Field data show that *T. conura* adapted to *C. heterophyllum* do not experience significant intrinsic egg and larval mortality on *C. heterophyllum* (Romstöck 1987; Romstöck-Völkl 1990). The only factors significantly reducing survival during development seem to be parasitoids and/or predators and bud abortion (Romstöck-Völkl 1990) but even in the presence of predators performance is much better on the original host with mean number of imagoes per infested flower = 5.41 (Nflowers = 78) (T. Diegisser and J. Johannesen, unpublished observations) vs. 3.67 in this study. The low number of surviving *T. conura* adapted to *C. heterophyllum* on *C. oleraceum* is likely not a consequence of increased competition in smaller *C. oleraceum* flower heads, as evidenced by larval capacity in Table 1. Also, Romstöck-Völkl (1990) calculated the receptacle surface needed per larvae in *C. heterophyllum* buds to be 6–8 mm2, which would allow 8–10 adult *T. conura* adapted to *C. heterophyllum* to develop in *C. oleraceum.* Thus, we conclude that the *T. conura* population adapted to *C. heterophyllum* is best adapted to *C. heterophyllum.* Furthermore, evolved avoidance mechanisms of *T. conura* adapted to *C. oleraceum* towards *C. heterophyllum* limits gene flow significantly, but data on performance within flower head are still missing.

**Table 1.**
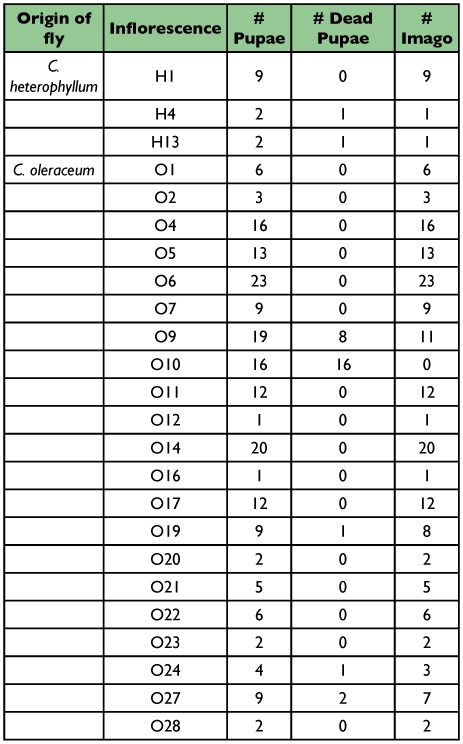
The number of pupae and imagos produced from successful oviposition on *Qrsium oleraceum* inflorescences for flies originating from *C. heterophyllum* and *C. oleraceum*.

Despite low performance of *T. conura* adapted to *C. heterophyllum* on *C. oleraceum*, the experiment showed genetic variation for survival on the wrong, low quality host. Significant within-population variability in performance has been found in many herbivore populations (see Via 1990), including tephritid host races (Craig et al. 1997). As selection on performance is most likely very strong, pre-adapted genotypes would spread rapidly and dominate the gene pool of *C. oleraceum* infesting flies within a few generations. Adaptation may be especially rapid when performance is determined by a few genes with large effects (Sezer and Butlin 1998). But why should *T. conura* shift to a low quality host plant? *Cirsium heterophyllum* and *C. oleraceum* have different temperature requirements and generally only co-occur in narrow contact zones. If the normal host is locally rare or becomes absent, oviposition into a novel host might be favoured despite poor larval performance. The experiment showed that, if forced, oviposition was attempted at low frequency on the wrong host. Indeed, mtDNA data point towards a peripatric origin of *T. conura* adapted to *C. oleraceum* (Diegisser et al. 2006a). Theoretical predictions that oviposition preference will shift towards less suitable hosts if hosts which provide a suitable larval environment become scarce (Levins and MacArthur 1969; Jaenike 1978) has been verified in empirical studies where alternative hosts were accepted if ‘high quality’ hosts were absent (Singer 1983; Etges and Heed 1987; Courtney and Forsberg 1988).

In conclusion, it has been demonstrated that the *T. conura* host race on *C. heterophyllum* have adapted physiologically to *C. oleraceum* in the course of a host shift. It is likely that low abundance of *C. heterophyllum* favoured the host shift to *C. oleraceum.*
